# BK Virus in Kidney Transplant Recipients: The Influence of Immunosuppression

**DOI:** 10.1155/2011/750836

**Published:** 2011-06-02

**Authors:** Katherine A. Barraclough, Nicole M. Isbel, Christine E. Staatz, David W. Johnson

**Affiliations:** ^1^Department of Nephrology, University of Queensland at the Princess Alexandra Hospital, Brisbane, QLD 4102, Australia; ^2^School of Pharmacy, University of Queensland, St Lucia, Brisbane, QLD 4072, Australia

## Abstract

The incidence of BK virus infection in kidney transplant recipients has increased over recent decades, coincident with the use of more potent immunosuppression. More importantly, posttransplant BK virus replication has emerged as an important cause of graft damage and subsequent graft loss. Immunosuppression has been accepted as a major risk for BK virus replication. However, the specific contribution of individual immunosuppressive medications to this risk has not been well established. The purpose of this paper is to provide an overview of the recent literature on the influence of the various immunosuppressant drugs and drug combinations on posttransplant BK virus replication. Evidence supporting the various immunosuppression reduction strategies utilised in the management of BK virus will also be briefly discussed.

## 1. Introduction

Advances in immunosuppressive therapies have markedly improved short-term kidney transplant outcomes. There has been a substantial decrease in acute rejection rates, and in most centres, 1-year graft survival now exceeds 95% [1–3]. In contrast, recent years have seen only minimal gains in long-term transplant outcomes [4]. Death-censored graft survival has only marginally increased [1–3], and life expectancy of kidney transplant recipients (KTxRs) remains markedly lower than that of the general population [5]. In large part, this is due to complications associated with life-long immunosuppression. 

Disease associated with BK virus (BKV) is one such complication. BKV is a small, ubiquitous, nonenveloped double-stranded DNA virus of the polyomavirus family. Its genome comprises early genes that code for the regulatory small and large T (LT) proteins and late genes that code for the viral capsid proteins (VP1, VP2, and VP3) and agnoprotein [6]. The genome also includes a noncoding control region (NCCR) that contains the origin of viral replication and the transcription and promoter sequences that control viral gene expression. 

Serological studies indicate that BKV infection has a worldwide adult seroprevalence rate of around 75% (range of 46–94% depending on the region studied) [6, 7]. Primary infection is usually asymptomatic, but BKV establishes latency in urinary epithelium [6–8]. While reactivation and urinary shedding occurs in 10% to 60% of immunocompetent individuals [7, 9, 10], markedly higher rates have been reported in immunocompromised individuals [11]. BKV appears to cause clinical disease only in individuals with changed or altered immune responses. This has been documented in pregnant individuals, those with human immunodeficiency virus-1 (HIV-1) infection or receiving chemotherapy, and in bone marrow and solid organ transplant recipients [12]. 

BKV infection in KTxRs has been described as causing several different manifestations. These include ureteric stenosis, haemorrhagic cystitis, transient renal dysfunction, and progressive renal impairment due to BKV-associated nephropathy (BKVAN) [6]. Of these, BKVAN is the most common and the most clinically significant because of its association with graft loss [13]. BKVAN was essentially a nonexistent entity in the 1980s and early 1990s, confirmed by a study that retrospectively reviewed biopsy slides of kidney transplant patients shedding decoy cells (cells in the urine that contain viral inclusions) between 1985 and 1996 [14]. However, its incidence has steadily increased in the subsequent years, with reports from recent decades describing incidence rates as high as 10% [15]. More importantly, BKVAN has emerged as an important cause of graft loss, reported in 0% to 80% of cases depending on immunosuppressive regimen employed, cohort size, timing of detection, and management strategy instituted [6, 13, 16]. 

Current knowledge regarding risk factors for BKVAN in the posttransplant period is extremely limited and inconsistent. A number of clinical and demographic factors have been associated with increased risk (Table 1) [17–35], but most have been only variably implicated and have limited predictive value [36]. More plausible is the notion that risk of BKVAN is dependent on the interaction of multiple risk factors [6], with a primary contribution from immunosuppression, and additional contributions from such donor, recipient, and viral factors as those tabulated.

## 2. Immunosuppression and BKV

Immunosuppression is the most significant and the only widely accepted risk factor for posttransplant BKV replication. This is largely because BKV associated disease is seen only in immunosuppressed populations, and because multiple studies have shown reductions in BKV replication following immunosuppression minimisation [6]. However, the relationship between BKVAN and immunosuppression remains poorly defined. Particularly, it remains unclear whether any particular agent can be specifically implicated, or whether overall potency of immunosuppression is responsible.

### 2.1. *In Vitro* Studies

Surprisingly, in addition to its immunosuppressive properties, cyclosporine has been shown to possess antiviral activity *in vitro* against herpes simplex virus [38], vaccinia virus [39], HIV-1 [40, 41], and hepatitis C virus [42–45]. Similarly, some studies have shown a suppressive effect of mycophenolic acid (MPA; the active drug moiety of mycophenolate mofetil (MMF) and enteric-coated mycophenolate sodium (EC-MPS)) on the *in vitro* replication of various herpes viruses [46], HIV-1 [47–49], and hepatitis B virus [50–52]. Based on these data, Acott et al. [53, 54] investigated the impact of cyclosporine and MPA on BKV replication using Vero E6 cells of green monkey origin infected with BKV (VJ isolate) when 70–90% confluence had been reached. Clinically utilised concentrations of cyclosporine were shown to not only inhibit the primary BKV infection peak, but also to inhibit BKV reactivation and NCCR gene rearrangements in a dose-independent manner [53]. The magnitudes of these observed effects were similar at all cyclosporine doses tested (200–12800 *μ*g/L). No antiviral effect of cyclosporine was seen on transformed virus or in cells with high-level infection (>10^9^ copies/mL). In a separate study, the same group showed a dose-dependent suppressive effect of clinically relevant concentrations of MPA (1–16 *μ*g/mL) on early and persistent BKV infection [54]. In contrast to cyclosporine, when added to culture at high doses (>8 *μ*g/mL), MPA was also able to inhibit high-level infection, and infection with NCCR rearranged virus. 

A concern with both of the above-mentioned studies is that all results were obtained from a Vero E6 monkey cell line. This raises the possibility of a cell-specific effect. To prove relevance of these findings to humans, a recent study investigated the effect of cyclosporine on BKV-(archetype strain-) infected human proximal renal tubular (HK-2) cells [55]. Cyclosporine (concentration range of 0.5–4 *μ*g/mL) was shown to dose-dependently reduce the expression of BKV LT and VP1 gene transcripts and BKV LT protein and to prevent the LT protein-regulated increase in early promoter activity. Interestingly, while actual data was not presented, this paper also reported that addition of tacrolimus (0.5–10 ng/mL) to culture media had no impact on LT antigen expression, suggesting against a calcineurin inhibitor class effect. 

Sirolimus has been shown to inhibit the expression of BKV LT antigen in both immortalized human renal cells and primary human renal tubular cells [56]. The effect was dose-dependent, observed across the range of 30 to 150 ng/mL. As stated by the study's authors', 30 ng/mL represents peak serum levels of sirolimus in transplant patients, while the higher concentrations may reflect tissue levels. In this study, use of sirolimus alone had no effect on BKV replication. However, its use in combination with leflunomide led to significant reductions in BKV DNA. 

*In vitro *steroid treatment has been shown to confer enhanced host cell permissivity for BKV infection. Moens et al. [57] found that treatment of BKV-infected Vero cells with dexamethasone increased the expression of viral transcripts (particularly the capsid proteins) up to 11-fold. DNA-protein binding studies showed that this effect was mediated via a steroid hormone response unit in the NCCR region of the BKV genome.

One study has investigated the *in vitro* effects of immunosuppressive medications on BKV-specific T cell responses [58]. Egli et al. [58] exposed peripheral blood mononuclear cells (PBMCs) from nonimmunosuppressed BK seropositive healthy volunteers to increasing concentrations of tacrolimus, cyclosporine, MPA, and sirolimus. PBMCs were then stimulated with BKV large T-antigen, and T-cell responses were measured via an interferon gamma- (IFN-*γ*-) linked ELISPOT assay. Both tacrolimus and cyclosporine were shown to cause dose-dependent inhibition of IFN-*γ* expression. Specifically, tacrolimus concentrations >6 ng/mL were shown to cause >50% inhibition of BKV-specific T cells, while concentrations of <3 ng/mL led to <30% inhibition. Inhibition by cyclosporine was >50% at concentrations of 1920 ng/mL and <30% at concentrations of <960 ng/mL. In contrast, addition of clinically relevant concentrations of MPA (up to 8 /mL) and sirolimus (up to 64 ng/mL) had no effect on BKV-specific T cell IFN-*γ* production. 

Of note, addition of increasing concentrations of sirolimus during T-cell expansion led to reductions in total cell count, consistent with the well-established anti-proliferative effect of this drug. This suggests that while sirolimus had no effect on T-cell activation, it was able to inhibit BKV-specific T-cell expansion. 

The above *in vitro* data suggest that cyclosporine, MPA, and sirolimus may be protective against BK replication, as compared to tacrolimus and corticosteroids. However, whether the postulated antiviral effects of these agents are sufficient to outweigh their immunosuppressive properties *in vivo* cannot be elucidated from these studies.

### 2.2. Clinical Studies

#### 2.2.1. Calcineurin Inhibitors, Antimetabolites, and Risk of BKV Replication

BKVAN was essentially an unknown entity in the era of cyclosporine-based immunosuppression, with increasing identification of BKVAN coinciding with inclusion of tacrolimus and MPA in immunosuppressive regimens [6]. This has led to the suggestion that these agents may be specifically responsible. In support of this, multiple retrospective and observational studies have demonstrated substantial increases in the risk of BKV replication in the context of tacrolimus and MPA use [14, 59, 60].  Table 2 details these studies. Of importance, however, in the first four of the studies tabulated [14, 20, 59, 60], conversion to tacrolimus occurred in response to episodes of rejection. This makes it likely that the development of BKVAN was preceded by overall intensification of immunosuppression. Additionally, it is possible that the renal injury arising from rejection episodes may have had a predisposing effect [61]. In the subsequent three studies tabulated [35, 61, 62], no information was provided as to why recipients were administered a particular calcineurin inhibitor or antimetabolite over the other. Given the general belief that tacrolimus and MPA have increased immunosuppressive potency compared to cyclosporine and azathioprine, use of these drugs may have been reserved for patients at higher immunological risk and were possibly dosed to achieve higher levels of immunosuppression. Consequently, the potential for indication bias limits any conclusions that may be drawn regarding drug-specific effects on BKV replication. 

Of note, while BKVAN was not observed in previous decades in those receiving cyclosporine-based immunosuppression, a more recent study of 321 KTxRs treated with cyclosporine, azathioprine, and prednisolone showed an incidence of BKVAN of 9.3% [63]. Given that no details regarding induction therapy or previous episodes of rejection were provided, it is difficult to evaluate in this study the influence of overall immunosuppressive burden on BKV replication. However, it does illustrate that BKVAN cannot be solely attributed to either tacrolimus or mycophenolate-based immunosuppression.

A number of studies have attempted to examine the relationship between BKV replication and immunosuppressive burden, as quantified by tacrolimus trough levels and MMF and prednisolone drug doses [20, 62, 64–67] (see Table 3). In three of these studies [64–66], an association was observed between higher tacrolimus trough concentrations and BKV replication. One of these studies [64] reported that MMF and prednisolone doses were similar between groups, while another described an association between prednisolone but not MMF dose and BKVAN [65]. In contrast, three studies have shown no influence of tacrolimus trough concentrations on BK replication [20, 62, 67]. Two reported no association between BKV and MMF dose [20, 67], while one reported no association between BKV and prednisolone dose [20].

Overall, these data perhaps suggest more prominent roles for tacrolimus and possibly prednisolone as predisposing agents for BK replication, compared to MPA. Indeed, the authors of the second study tabulated concluded that reducing tacrolimus and prednisolone doses while maintaining MMF might be the most appropriate approach for treatment of BKVAN [68]. However, while it is generally accepted that tacrolimus trough concentrations are a reasonable surrogate measure of drug exposure [69], both MPA and prednisolone are drugs with high between-subject pharmacokinetic variability leading to poor relationships between drug doses and plasma concentrations [70–72]. Similar doses of these drugs in those with and without BK replication cannot be equated with similar drug exposure. As a result, it is impossible to establish from these studies the individual contribution of MPA and prednisolone on BKV replication or to establish whether variable MPA and prednisolone exposure between groups may have confounded the data pertaining to tacrolimus concentrations.

An open-label, prospective, single-centre randomized controlled trial (RCT) of 200 *de novo *adult KTxRs provides the best evidence to date regarding the impact of the individual calcineurin inhibitors and antimetabolites on BK replication [73]. This study randomly assigned recipients to tacrolimus or cyclosporine, administered in combination with azathioprine and tapering doses of prednisolone. MMF (1 g twice daily) was substituted for azathioprine in those considered to be at high immunological risk or with a history of gout (49% in the tacrolimus group and 35% in the cyclosporine group). No difference was observed in the incidence of viruria in recipients assigned to cyclosporine compared to tacrolimus (36% versus 31%; *P* = .61) or in recipients who received azathioprine compared to MMF (33% versus 38%; *P* = .52). Similarly, there was no difference in viraemia with tacrolimus compared to cyclosporine (12% versus 11%; *P* = 1.0) or with azathioprine versus MMF (13% versus 9%; *P* = .46). While viruria was highest with the combination of tacrolimus and MMF (46%) and lowest with cyclosporine and MMF (13%) (*P* = .005), viraemia tended to be highest with cyclosporine and azathioprine (15%) and lowest with cyclosporine and MMF (4%) (*P* = .27). These data fail to demonstrate a single role of any particular CNI or antimetabolite in promoting BKV replication. The finding of the highest incidence of BK viruria with tacrolimus and MMF does suggest that this combination may be the most permissive for BKV replication. However, it should be noted that cyclosporine lowers MPA concentrations while tacrolimus does not [70], raising the possibility of confounding due to higher MPA exposure in the tacrolimus arm of the trial (MPA concentrations not measured). Further, those with both transient and sustained viruria were included in this analysis. Given that transient viruria can be observed in a variety of individuals including healthy donors and nonkidney solid organ transplant recipients, this data may be misleading with regard to the influence of immunosuppression on clinically significant BKV replication. The relative effects of cyclosporine versus tacrolimus on BKVAN are currently being assessed as a secondary end-point of the DIRECT (Diabetes Incidence after Renal transplantation: NEoral C-2 monitoring versus Tacrolimus) trial, a randomized, six-month, open label, international multicentre trial in which 682 patients who received kidney transplants were treated with either cyclosporine microemulsion formulation or tacrolimus to prevent organ rejection [74].

#### 2.2.2. Steroids and Risk of BKV Replication

Use of pulse steroids as treatment for acute rejection has been independently associated with BKV replication and BKVAN [21]. However, as discussed above, it is difficult to differentiate whether BKVAN following rejection results from augmented immunosuppression or from a drug-specific effect. There has been only limited study of the role of maintenance steroid therapy in promoting BKV replication. In a nonrandomized prospective study of 120 KTxRs of whom 71 were maintained on steroids and 49 were treated with early steroid withdrawal, Dadhania et al. [67] identified steroid maintenance therapy as an independent risk factor for BKV replication (odds ratio 8.3 (95% CI 2.1, 32.7); *P* = .003)). A retrospective single-centre study of 213 kidney and 14 pancreas-kidney transplant recipients found a lower incidence of BKVAN in those that had steroids ceased early or withdrawn, as compared to those that continued on steroids (0% versus 3.5%; *P* < .05) [75]. A US OPTN database review of 48,292 KTxRs [35] reported that the risk of treatment for BKV replication was increased in those discharged on maintenance steroids versus those that were not (adjusted hazards ratio 1.16 (95% CI 1.02, 1.31); *P* = .0237)). None of these studies reported on whether immunological risk status and immunosuppression target levels differed between groups. However, the study of Dadhania et al. [67] used multivariable logistic regression analysis to account for the effects of antihuman thymocyte globulin (ATG) induction, tacrolimus trough levels, tacrolimus and MMF dose and acute rejection, while the database review [35] fitted a Cox proportional hazards model to account for a very large number of possible confounding variables.

#### 2.2.3. Calcineurin Inhibitor-Free, Mammalian Target of Rapamycin (mTOR-) Based Regimens and Risk of BKV Replication

BKVAN has been uncommonly observed in patients receiving calcineurin inhibitor-free or mTOR-based regimens. A small case series reported the development of BKVAN in 3 KTxRs maintained on sirolimus, prednisolone, and MMF [76]. None had been previously exposed to calcineurin inhibitors or experienced prior rejection. Two had received interleukin-2 antagonist induction therapy, while one had received thymoglobulin. A retrospective study reported nine cases of BKVAN in 344 kidney and 34 pancreas-kidney transplant recipients treated with sirolimus-based immunosuppression (cyclosporine-sirolimus in 6 recipients, tacrolimus-sirolimus in 1 recipient, MMF-sirolimus in 1 recipient, and cyclosporine-MMF-sirolimus in 1 recipient) [77]. Eight of nine patients had been previously exposed to ATG, while 3 had experienced acute rejection. In the US OPTN database review [35], 5380 of 48,292 KTxRs were discharged on mTOR-based immunosuppression, of whom 83 (1.74%) received treatment for BKVAN within 2 years of transplant. Multivariable analysis showed a reduction in risk of treatment for BKV replication with use of an mTOR at discharge, as compared to no mTOR use (adjusted hazards ratio 0.69 (95% CI 0.54, 0.89); *P* = .0048)).

#### 2.2.4. Lymphocyte Depleting Therapy and Risk of BKV Replication

The majority of studies have shown an increase in BKV replication following use of lymphocyte depleting agents for induction or treatment of rejection. This is not surprising given the immunosuppressive potency of these agents. In a study of 120 KTxRs, multivariable analysis showed an independent influence of ATG induction on risk of BKV replication (odds ratio 5.83 (95% CI 1.60, 21.35); *P* = .008) [67]. Similarly, in the retrospective study of 344 kidney and 34 pancreas-kidney transplant recipients mentioned above [77], multivariable analysis correlated exposure to ATG for either induction or rejection treatment with a higher incidence of BKVAN (3.53% versus 1.44%; *P* = .048). In a retrospective analysis of renal biopsy and urine cytology samples from 880 KTxRs, use of ATG for induction exerted an independent risk for developing both high level viruria and BKVAN [61]. In a prospective study of 78 KTxRs, those with viraemia were more likely to have received antirejection treatment with ATG than those without evidence of BKV replication (60% versus 20%; *P* = .008) [21]. Interestingly, in the US OPTN database review [35], induction therapy with thymoglobulin was associated with an increased risk of treatment for BKV (adjusted hazards ratio 1.42 (95% CI 1.24, 1.63); *P* < .0001), but induction therapy with ATG had no independent effect (adjusted hazards ratio 01.19 (95% CI 0.73, 1.95); *P* = .4792).

## 3. Immunosuppression Reduction as a Treatment Strategy for BKV Replication

To date, there are no antiviral drugs available with specific activity against BKV [37]. While various therapies have been tried, including cidofovir [78, 79], intravenous immunoglobulin [80], and leflunomide [81], success has been variable and none have been appropriately studied in a randomized controlled clinical trial [37]. Consequently, reduction of immunosuppression remains the mainstay of treatment, despite the increased risk of immunological allograft damage associated with this approach. Multiple strategies are currently utilised, including reducing or ceasing antimetabolite therapy, lowering calcineurin inhibitor target concentrations, switching from tacrolimus to cyclosporine, and conversion from calcineurin inhibitor to sirolimus [13, 82]. However, as outlined in a recent systematic review [83], published reports on these protocols have yielded mixed results, and no randomized controlled trials have compared one strategy with another. 

Of note, in more recent times, many centers have instituted routine screening of urine or blood for BKV DNA. Such programs have reported significant improvements in graft outcomes, possibly due to early detection of viral presence and reduction of immunosuppression prior to the onset of graft damage [17, 21, 84, 85].

## 4. Conclusions

Immunosuppression is clearly a major risk factor for BKV. This is evidenced by the increase in viral replication observed in immunosuppressed populations and the decrease in viral replication that follows immunosuppression reduction. To date, however, there is no conclusive evidence that any one drug has specific influence over another in regard to the development of posttransplant BKV infection.

Cyclosporine, mycophenolate, and sirolimus have all been shown to possess antiviral activity against BKV *in vitro*. Alternatively, *in vitro* data suggest that tacrolimus has no inhibitory potential, while corticosteroids may in fact enhance BKV replication. However, whether these characteristics are sufficient to counteract or exacerbate the immunosuppressive properties of these drugs *in vivo *has not been confirmed in clinical trials. 

In KTxRs, BKV infection has been observed under all combinations of immunosuppression. This illustrates that no one drug is either necessary or sufficient. While multiple studies have associated tacrolimus and MMF use with increased risk, the majority of these were retrospective or observational in nature and confounded by variable and uncontrolled use of additional immunosuppression. Only one RCT has addressed this issue. This showed no independent influence of either tacrolimus or cyclosporine, nor any independent effect of either azathioprine or MMF. However, again, results were likely cofounded by variable immunosuppressive cotherapy exposure in the two arms of this trial. A small number of studies have shown lower incidence of BKV replication under steroid minimisation, CNI-free, or mTOR-based regimens. However, given that such protocols are often reserved for lower immunological risk patients requiring lower total doses of immunosuppression, little can be interpreted from this data.

There is general consensus that reduction of immunosuppression is appropriate for management of significant BKV replication. Further, it appears that early detection and intervention are important in preventing irreversible graft damage. However, there has been limited comparison of the various approaches, with the result that there is no good evidence to support one strategy over another. 

For the future, prospective trials specifically designed to address the impact of the various immunosuppressive agents on risk of BKV replication are required. To prevent confounding of results due to variable cotherapy exposure, it is vital that these studies incorporate proper immunosuppression exposure measures (e.g., MPA drug concentration measurements). Additionally, assays are becoming available that allow direct assessment of immune cell function [86]. Incorporation of these measures could prove invaluable in distinguishing postulated antiviral effects of the various drugs from the influence of drug-induced immunosuppression.

## Figures and Tables

**Table 1:** Factors other than immunosuppression associated with increased risk of posttransplant BKV replication.

Risk factors	Reference
Older age	[20, 35]
Male gender	[20, 35]
Caucasian race	[18, 35]
Diabetes mellitus	[18]
Deceased donor	[19]
Greater HLA mismatches	[20–22, 35]
Absence of HLA-C7 in donor and recipient	[23]
Acute rejection	[18, 21, 35]
Recipient seronegativity	[25, 33]
Recipient seropositivity	[26]
Donor seropositivity	[23, 27, 28]
High donor antibody titre	[23]
Poor cellular immune response	[30–34, 37]
Longer cold ischemic time	[35]
Use of ureteral stent	[35]
Delayed graft function	[35]
More recent transplant year	[35]
Lower centre transplant volume	[35]

BKV: BK virus; HLA: human leukocyte antigen.

**Table 2:** Clinical studies examining the influence of the calcineurin inhibitors and antimetabolites on risk of BKV replication.

Study number	Number of participants	Study design	Primary maintenance immunosuppressive therapy*	Manifestation of BKV	Results	Reference
1	161 KTxRs	Retrospective	73% CyA, MMF, pred; 27% Tac, MMF, pred	8 with BKVAN	7 of 8 recipients with BKVAN receiving Tac, MMF, and pred	[59]

2	70 KTxRs	Retrospective	100% CyA, MMF, pred	5 with BKVAN	BKVAN followed switch from CyA to Tac in all 5 cases	[14]

3	483 KTxRs	Retrospective	Not specified	100 with viruria; 5 with BKVAN	BKVAN followed switch from CyA to Tac in all 5 cases	[60]

4	1276 renal biopsies	Retrospective	45% CyA and pred; 11% Tac and pred; 30% CyA, MMF, and pred; 8% Tac, MMF, and pred; 5% CyA and Aza; 3% CyA, SLM, and pred	7 with BKVAN	4 of 7 recipients with BKVAN were receiving Tac, MMF, and pred; use of this combination associated with an 8-fold higher incidence and 13-fold higher risk of BKVAN (multivariate odds ratio of 12.7 (95% CI 2.1–7.8))^#^	[20]

5	880 KTxRs	Retrospective	75% CyA, 16% Tac, 7% SLM; 64% Aza, 34% MMF; “Almost all” pred	129 with viruria; 39 with BKVAN	Both Tac and MMF independently associated with increased risk of high-titre viruria (hazard ratios of 4.9 (95% CI 3.1–7.3) and 4.6 (95% CI 3.1–6.7) for the 2 drugs, resp.), and for BKVAN (hazard ratios of 3.3 (95% CI 1.5–7.6) and 3.5 (95%CI 1.6–7.5), for the 2 drugs, resp.).	[61]

6	32 KTxRs	Prospective observational	59% CyA, MMF, pred-based; 41% Tac, MMF, pred	20 with viruria^*∧*^; 23 with viraemia	Higher rate and risk of viremia with Tac as compared to CyA (77% versus 33%, *P* = .03; relative risk for viraemia with tacrolimus use 2.3 (95% CI 1.1–4.9))	[62]

7	48,292 KTxRs	Retrospective	20% CyA, 76% Tac, 4% no CNI; 1% Aza, 82% MMF, 17% no antimetabolite	1474 treated for BKV replication	Lower adjusted hazard ratio for treatment of BKV replication with CyA-based as compared to Tac-based immunosuppression (0.53 (95% CI 0.45, 0.63); *P* < .0001); trend towards reduced risk with Aza compared to MMF (0.42 (95% CI 0.17, 1.01); *P* = .0517).	[35]

*Refers to initial maintenance regimen employed at time of transplantation; not necessarily the regimen employed at time of diagnosis with BKV replication.

^#^For the analysis relating to the influence of immunosuppression on BKVAN, the KTxRs with BKVAN were compared to a control group (*n* = 42; matched for transplant date and baseline immunosuppression) rather than to study cohort as a whole.

^*∧*^Includes recipients in whom viruria and viraemia were simultaneously detected.

Aza: azathioprine; BKV: BK virus; BKVAN: BKV-associated nephropathy; CyA: cyclosporine; KTxRs: kidney transplant recipients; MMF: mycophenolate mofetil; pred: prednisolone; SLM: sirolimus; Tac: tacrolimus.

**Table 3:** Clinical studies examining the relationship between BKV replication and drug exposure.

Study number	Number of participants	Study design	Immunosuppression exposure measures	Manifestation of BKV	Results	Reference
1	38 pancreas Tx recipients	Retrospective	Tac C_0_ concentrations, MMF, and pred dose	4 with viruria	Tac C_0_ higher in those with viruria at 1, 3 and 12 months post-tx (22 ± 3.1 versus 16 ± 0.9 ng/mL, *P* = .03; 26 ± 4.8 versus 14 ± 1.0 ng/mL, *P* = .0005; 19 ± 0.6 versus 12 ± 0.7 ng/mL *P* = .0008); no influence of MMF or pred dose	[65]

2	33 KTxRs with BKVAN, 66 matched controls	Retrospective	Tac C_0_ concentrations, MMF, and pred dose	33 with BKVAN	Higher Tac C_0_ and prednisolone dose associated with increased risk of BKVAN (OR 1.3. 95% CI 1.02–1.7; *P* = .03; and OR 1.22, 95% CI 1.04–1.4; *P* = .02, resp.); no influence of MMF dose	[66]

3	575 KTxRs	Retrospective	Tac C_0_ concentrations	32 with BKVAN	Higher Tac C_0_ associated with higher incidence of BKVAN (10.5% versus 2.5%, *P* < .09001)	[64]

4	7 KTxRs with BKVAN, 42 matched controls^#^	Retrospective	Tac C_0_ concentrations, MMF, and pred dose	7 with BKVAN	No influence of Tac C_0_, MMF, or pred dose on BKVAN	[20]

5	32 KTxRs	Prospective observational	CyA and Tac C_0_ concentrations	20 with viruria*; 23 with viraemia	No influence of CyA or Tac C_0_ on viruria, viraemia or BKVAN	[62]

6	120 KTxRs	Prospective observational	Tac C_0_ concentrations, tac, and pred dose	37 with viruria	No influence of Tac C_0_ or tac or pred dose on viruria	[67],

*Includes recipients in whom viruria and viraemia were simultaneously detected.

^#^Matched for transplant date and baseline immunosuppression.

BKV: BK virus; BKVAN: BKV associated nephropathy; CI: confidence interval; CyA: cyclosporine; KTxRs: kidney transplant recipients; MMF: mycophenolate mofetil; OR: odds ratio; pred: prednisolone; Tac: tacrolimus.
